# Mechanistic analysis elucidating the relationship between Lys96 mutation in *Mycobacterium tuberculosis *pyrazinamidase enzyme and pyrazinamide susceptibility

**DOI:** 10.1186/1471-2164-16-S2-S14

**Published:** 2015-01-21

**Authors:** Chakshu Vats, Jaspreet Kaur Dhanjal, Sukriti Goyal, Ankita Gupta, Navneeta Bharadvaja, Abhinav Grover

**Affiliations:** 1 Department of Biotechnology, Delhi Technological University, New Delhi, India - 110042; 2 School of Biotechnology, Jawaharlal Nehru University, New Delhi, India - 110067; 3 School of Bioscience and Biotechnology, Banasthali University, Tonk, Rajasthan, India - 304022

**Keywords:** Tuberculosis, Pyrazinamide, pyrazinamidase, mutations, drug resistance, molecular dynamics simulations

## Abstract

### Background

Pyrazinamide (PZA) is one of the most effective first line treatments against tuberculosis disease. The drug generally has bacteriostatic action. It also acts on bacterial spores which eliminates the chances of resurfacing of the infection. However, in recent years there has been a major increase in the occurrence of drug resistant bacterial strains. Resistance against PZA is caused by mutations in pyrazinamidase (PncA) protein which is the activator of the prodrug PZA. In the present study, we have tried to gain insights into the mechanism by which resistance develops due to K96R mutation occurring in the PncA catalytic region

### Results

The binding cavity analysis showed an increase of 762.3 Å^3 ^in the volume of the mutant protein. Docking studies revealed that PZA has a greater binding affinity for the native protein in comparison to the mutant protein. Molecular dynamics simulations further showed the role of flap region, which is present in PncA protein, in development of resistance to the drug.

### Conclusion

The residues of flap region acquire more flexibility in mutant form of protein and thus move away from the active site. This leads to weak binding of the drug to the target residues which might interfere with the activation of the drug to functional form thereby giving rise to drug resistant bacterial strains.

## Background

One of the major concerns being faced by the world today is tuberculosis disease (TB). About one third of the world population is suffering from the infection caused by *Mycobacterium tuberculosis *[1]. It was estimated, that in 2012, around 8.6 million people developed TB and approximately 1.3 million people died because of this disease [2]. The emergence of drug-resistant bacterial strains is one of the main causes for the current spread of TB [3]. MDR TB or multi drug resistant tuberculosis is caused when resistance is developed for two or more first line drugs. Treatment of drug-resistant tuberculosis is hampered by poor efficacy and high toxicity of the second-line drugs [4]. Continuous efforts are being made worldwide to find out some novel protein targets against which new effective drugs can be designed [5,6].

Various new approaches have come up for the designing of drugs for such complex diseases. One of the strategies includes the development of small molecule which could either inhibit multiple sites on the same target or inhibit altogether different protein targets. Some previous work done in our lab to check pathologies associated with the Alzheimer's disease illustrates this well. In one of the experiments two small molecular compounds were proposed, which had inhibitory activity against two of the important targets associated with Alzheimer's- β-amyloid cleavage enzyme and acetylcholine esterase (AChE) [7]. In another work, compounds having inhibitory activity against two different sites of AChE enzyme were identified [8]. Molecular dynamics (MD) simulations have further made it easier to investigate the dynamic behavior of proteins using *in silico *techniques. It drastically narrows down the investment of time, cost and effort by limiting the number of cases for which experimental verification needs to be carried out [9]. MD simulations have made it possible to study conformational changes that might occur in the three dimensional structural of proteins upon any change in the sequence. Therefore the effect of mutations occurring in a protein can be elucidated in detail using this computational approach. This can further be used to investigate the mechanism that leads to resistance of drugs in certain diseases. This insight into the mode of action of drug with respect to changes that occur in the three dimensional structure of protein can then be used to design drugs effective against both wild type and mutant isoforms of the target associated with the disease.

Pyrazinamide (PZA), a derivative of nicotinamide is one of the most imperative first-line drug treatments against tuberculosis [10]. PZA is significantly used in MDR tuberculosis in combination with isoniazid, rifampicin and ethambutol in regimens. The most potent action of the drug is against the semi-dormant bacilli in an acidic environment, which cannot be treated with most other drugs and thus helps in shortening the chemotherapy period [11].

However, PZA is a pro-drug and acts only when metabolized to pyrazonic acid (POA). Pyrazonic acid is derived by the action of an enzyme called pyrazinamidase, encoded by pncA gene. Mycobacterial pyrazinamidase (MtPncA) is constitutively expressed in the cytoplasm and plays a pivotal role in the activation of pyrazinamide[12]. Only after conversion of PZA into pyrazinoic acid by this enzyme the drug becomes potent. The bactericidal effect of this drug is attributed to destabilization of membrane potential and changes in the transport function [13-15].

Emergence of drug resistant strains of tuberculosis is one of the major concerns being faced by both developed and developing countries. Several mutations have been identified in Mtb proteins making them resistant to current drug therapies. Mutations in pncA play a major role in PZA resistance in *M. tuberculosis *[16-20]. The loss of PZA activity has been attributed to non-synonymous mutations in the gene pncA. Studies have shown that mutations, based on their location, were able to either decrease the activity several folds or confer resistance to PZA [19,21-23].

Several studies have been performed to analyze the resistance mechanism of the drug. One such study analyzed three mutations namely D8G, S104R and C138Y. Study involved analysis of binding pocket, molecular docking studies and molecular dynamics simulations. This helped in understanding the interaction pattern of the drug with the enzyme and three-dimensional (3D) conformational behaviour of native and mutant PncA. The analysis predicted that the inhibition of the activity of the drug is due to change in conformation of the enzyme. According to the results, the binding cavity of the enzyme becomes rigid due to these mutations, which hampers the binding of the conversion of PZA to its active form. The major reason for rigidity is excessive hydrogen bonding between PZA binding cavity residues and their neighboring residues [24].

The 3D-structure of PncA protein is available in PDB (PDB ID: 3PLI). The structure was determined at a resolution of 2.2Å using PhPncA by molecular replacement [25]. The protein consists of 185 amino acid residues as revealed by X- ray crystallography. Six stranded parallel beta sheets and four alpha helices pack together to form alpha/beta domain. Site directed mutagenesis based experiments revealed Asp 8, Lys 96 and Cys138 to be the pivotal residues involved in catalysis and Asp 49, His 57 and His 71 as the metal ion binding residues. Mutations at these residues greatly affect the function of PncA enzyme [19,21,25,26]. These active residues are mostly present in the alpha- 3 helix which lies in the N-terminal region of the protein [25].

One of the most prominent characteristic of the mutations identified from the data obtained from clinical isolates is their diversity. These mutations range from missense mutations to insertions and deletions and are spread throughout the pncA gene [16,27,28]. Since the drug is metabolized in acidic conditions, it is cumbersome to obtain reliable interpretation of data on resistance of *M. tuberculosis *to the drug. Therefore, PCR amplification and DNA sequencing of pncA is an approach of interest as it allows rapid identification of the pncA mutations that may be involved in PZA resistance. This information can be used to study the correlation between structure and function i.e. to study the effects of mutation on the function of the enzyme. Mutational analysis thus can be used to assess PZA susceptibility.

In this study, a novel mutation Lys-96-Arg (K96R) has been considered. This mutation was revealed by deep sequencing via NGS (Next Generation sequencing) in the pncA gene of 26 random Multi Drug Resistant Tuberculosis patients of West African origin residing in USA. 9 out of 26 isolates were carrying this mutation making them resistant to pyrazinamide and in one case to all 1^st ^and 2^nd ^line drugs. This mutation lies in the active center of the protein and thereby is of utmost importance. Considering the ratio 9/26, the mutation seems to be quite prevalent. Since, tuberculosis is one of the most widespread disease, counteracting the causes of drug resistance via computational studies is a good choice [29].

Here, we have tried to answer the questions recently raised by some researches. First, what are the structural changes produced by this mutation? Second, what is the mechanism by which resistance to this drug develops due to this mutation? In the present work, we studied the relationship between structural and physico-chemical characteristics of PncA, with K96R mutation in the active site, and the enzymatic function to understand PZA resistance in *M. tuberculosis*. Critical understanding of these mechanisms allows the development of robust and efficient molecular diagnostic tests and provides a platform for the development of new drugs. Further, knowledge of these mechanisms can help in developing precautionary measures to curb the development of resistance [30].

In this study, we have performed several analyses to understand the mechanism of resistance including docking studies, binding pocket analysis and molecular dynamics of both native and mutant PncA. The MD simulations are known to provide the dynamic conformational changes in the interaction of protein and ligand [31]. The results obtained inferred that the drug resistance could be due to the alteration in 3-D conformation of the protein by mobility of the binding residues in the mutant. The comparison of dynamic properties of native and mutant protein was done on the basis of molecular flexibility of the binding residues, the network of bonds and the fluctuations observed in them. The main objective of our work was to understand the molecular mechanism of PncA resistance at molecular level which causes PZA resistance in TB patients.

## Materials and methods

### Structure of protein and ligand

We retrieved the native PncA protein structure (PDB: 3PL1) from Protein Data Bank (PDB) [25,32]. The pdb structure contained a Fe^2+ ^ion surrounded by one aspartate and three histidines in the substrate binding cavity along with three water molecules. The three most important functional residues include Asp 8, Lys 96 and Asp 138. In order to perform further analysis the crystallized water molecules were removed using Accelyrs Viewerlite 5.0 [33]. The 3-D structure of the drug molecule PZA was taken from drug bank in .sdf format [34]. Since the mutant structure was not available in the database, a point mutation was introduced *in silico *at 96^th ^position in the native structure of the protein to create the mutant form.

### Binding cavity analysis

The size of the binding cavity is an important measure in mutational studies. To compare the binding cavities of native and mutant proteins, we used CASTp server [35]. CASTp algorithm is based upon recent developments in Computational Geometry. It has certain advantages which include, 1) analytical identification of pockets and, 2) precisely defined boundary between solvent and pocket, and 3) calculated parameters does not make use of dot surface or grid points and are rotationally invariant. The server uses weighted Delaunay triangulation and alpha complex for shape measurements. It generates information about surface accessible pockets and interior inaccessible cavities for a given protein. It calculates the volume and area of each cavity in solvent accessible surface (SA, Richards' surface) and molecular surface (MS, Connolly's surface). Number, area and circumference of mouth pockets are also calculated. It requires the protein .pdb structure and the results are displayed in Jmol. We used the default values for all the calculations using this server. A value of 1.4 Å was used as probe radius. The results were validated using another program, MOLE 2.0 [36].

### Protein ligand interaction

Receptor-ligand interaction analysis was performed for native and mutant PncA with PZA. In order to carry out this docking study, we used the Glide module of Schrodinger [37,38]. The protein structure was prepared using Schrodinger's protein preparation wizard [39,40]. In this process hydrogen were added, bond lengths were optimized, disulfide bonds were created, terminal residues were capped and selenomethionines were converted to methionine. Flexible docking protocols were used in which both protein and ligand were kept flexible. PZA was prepared using LigPrep [41]. It generated all possible chiral, stereochemical and ionization variants of the ligand.

A grid was generated around the active site residues of the protein molecule using Glide module of Schrodinger. The grid was generated around Asp8, Phe13, Asp49, His51, His57, Phe58, Gly60, Trp68, His71, Lys96, Ile133, Ala134, Thr135, and Cys138. Extra precision docking was then performed for native and mutant proteins with PZA.

Total ligand-receptor interaction energy along with its components like van der Waals energy and electrostatic energy was also calculated. The electrostatic energy gives a measure of how a molecule interacts with another nearby molecule. The two molecules interact with each other through their mutual electrostatic interactions. The negative value of electrostatic energy indicates better interaction and vice versa. Since hydrogen bonds and van der Waals contacts depend on complementary surfaces, these surfaces must be able to pack closely together, creating many contact points allowing charged atoms to make electrostatic bonds. Thus, polar interactions and van der Waals contribute to the dynamic stability of the ligand-receptor complex [42].

The molecular docking results obtained were cross checked using PatchDock and SwissDock [43-45].

### Molecular dynamics simulations of the docked complexes

In order to investigate the mechanism of resistance in the docked complexes, molecular dynamic study was performed in the presence of an explicit fully hydrated solvent model using triclinic boundary. The simulations were performed using Desmond Molecular Dynamics module of Schrodinger, with Optimized Potentials for Liquid Simulations (OPLS) all-atom force field 2005 [46,47]. The complexes were prepared before simulation by addition of hydrogen atoms followed by optimization, capping of end terminals, conversion of selenomethionine to methionine and generation of disulphide bonds using the protein preparation wizard. Prepared protein-ligand complexes were then solvated with SPC water model in a triclinic periodic boundary box. To prevent interaction of the protein complex with its own periodic image, the distance between the complex and the box wall was kept 10 Å. Energy of the prepared systems was minimized for 5000 steps using steepest descent method or until a gradient threshold of 25kcal/mol/Å was achieved. It was followed by L-BFGS (Low-memory Broyden- Fletcher- Goldfarb Shanno quasi-Newtonian minimiser) until a convergence threshold of 1 kcal/mol/Å was met. For system equilibration, the default parameters in Desmond were applied. The equilibrated systems were then used for simulations at a temperature of 300 K and a constant pressure of 1atm, with a time step of 2fs. For handling long range electrostatic interactions Smooth Particle Mesh Ewald method was used whereas Cutoff method was selected to define the short range electrostatic interactions. A cut-off of 9 Å radius was used.

## Results and discussion

Continuous emergence of new mutations associated with drug resistance in *Mycobacterium tuberculosis *is posing a great danger to the conventional therapies. The methods need to be revised according to the current situation, which requires understanding of the mechanisms of resistance. In order to identify one such mechanism we have tried to analyze the mutation K96R occurring in the active site of *Mycobacterium tuberculosis*. Various analyses have been done in order to understand the mechanism at molecular level.

### Preparation of mutant protein

Since the structure of mutant protein was not available in the database, *in silico *mutation was created at position 96 by replacing lysine with arginine. In order to allow the protein to rearrange the backbone and the side chains of the mutated residue and the nearby amino acids so as to reach the conformational state with no steric clashes and minimum internal energy, it was subjected to MD simulation for 15 ns. RMSD of each frame in reference to the first frame was plotted against the simulation time to comment on the equilibrium state of the mutated protein. Initially an RMSD of 4Å was observed for the first 6 ns after which an almost stable trajectory was obtained. The coordinates of all the frames within the most stable time frame, i.e. from 6 to 15 ns, were averaged to compute a structure representing the equilibrated state of the mutated protein. This representative average structure was used for further study.

### Binding cavity analysis

**Figure 1:**
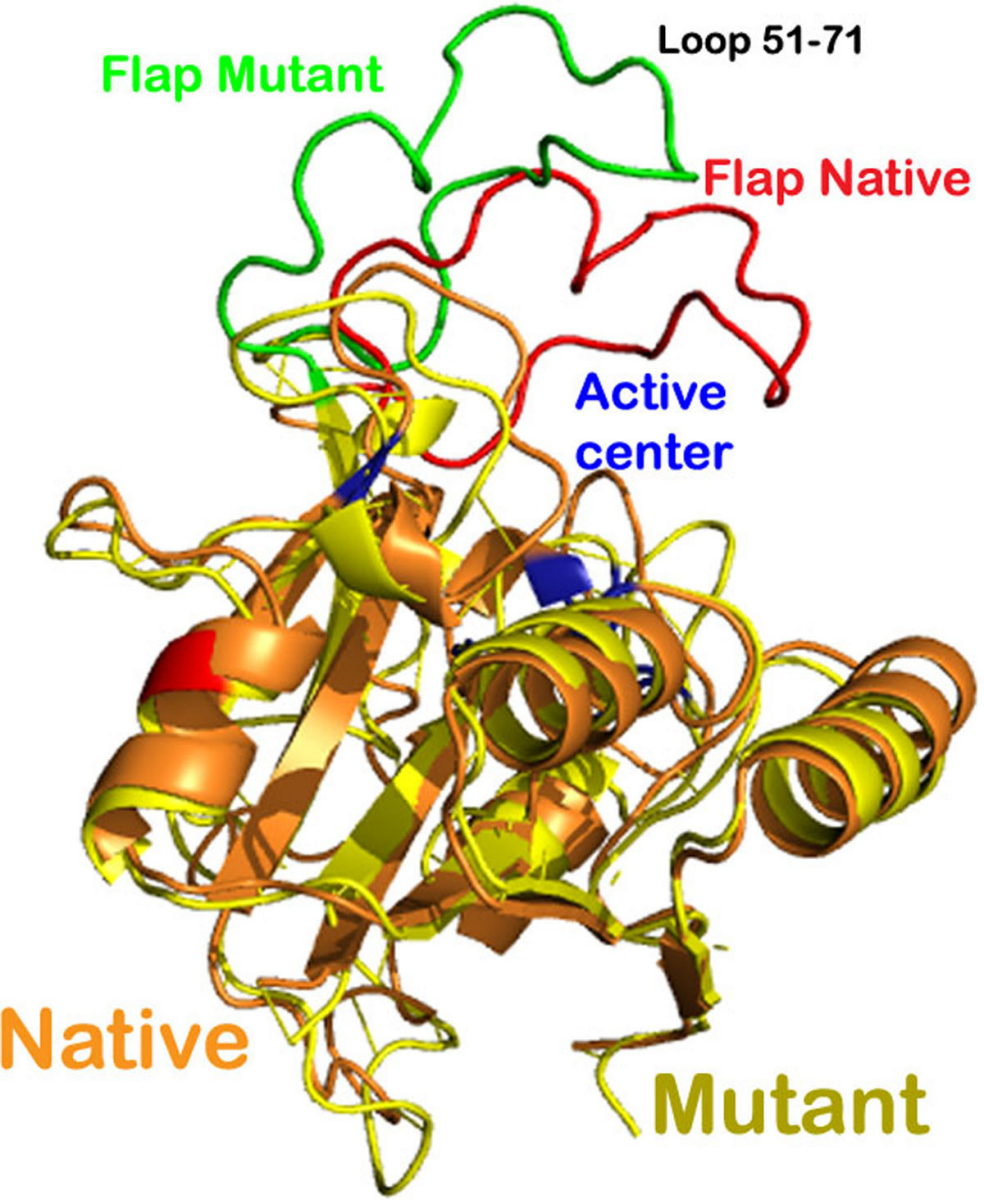
**Ribbon representation of the superimposition of structures of the native and mutant *M. tuberculosis *PncA protein**.

Binding pocket analysis is an important measure to identify the impact of mutation on the cavity. The pocket volume for both native and wild type were calculated using CASTp server. The structures obtained after molecular dynamics were used for the calculation of the pocket volume. The volume of the cavity for native PncA protein was 551.9 Å^3 ^and that of mutated form was 1314.2 Å^3^. A considerable difference can be seen in the two volumes (Figure 1). The increase in volume affects the binding of PZA within active cavity thereby decreasing the stability of the ligand. In order to further evaluate the behavior of the mutant, we performed docking and molecular dynamics simulation studies.

The change in the cavity size was further validated using MOLE 2.0. A significant increase in the active site volume was observed for the mutant protein with this program as well.

### Interaction analysis between receptor and ligand molecule

Previous studies on PncA have shown the significance of van der Waals interactions in binding to PZA. Binding energy calculations provide information on both van der Waals and electrostatic energy of the complex. The value of van der Waals energy and electrostatic energy for native complex calculated using Glide was found to be -17.16 kcal/mol and -10.27 kcal/mol respectively. The glide energy or the total ligand-receptor energy was found to be -27.43 kcal/mol for the native PncA and PZA complex. On the contrary, there was a significant difference in the values for the complex with mutated protein. The values for van der Waals, electrostatic and glide energy were -10.86, -13 and -23.43 kcal/mol respectively (Table 1). As indicated by the total energy, PZA in complex with mutated PncA was less stable in comparison to PZA docked with native PncA. Hydrogen and hydrophobic interactions also play a major role in making the binding stable. The interaction plots for these two types of interactions were generated using Ligplot [48].

**Table 1:** Scores from docking analysis of native and mutant protein

Protein	Docking Score (kcal/mol)	Glide Energy (kcal/mol)	Electrostatic energy (kcal/mol)	Vander waal Energy (kcal/mol)
Native	-4.025	-27.43	-17.16	-17.16
Mutant	-3.77	-23.434	-13.0	-10.86

**Figure 2:**
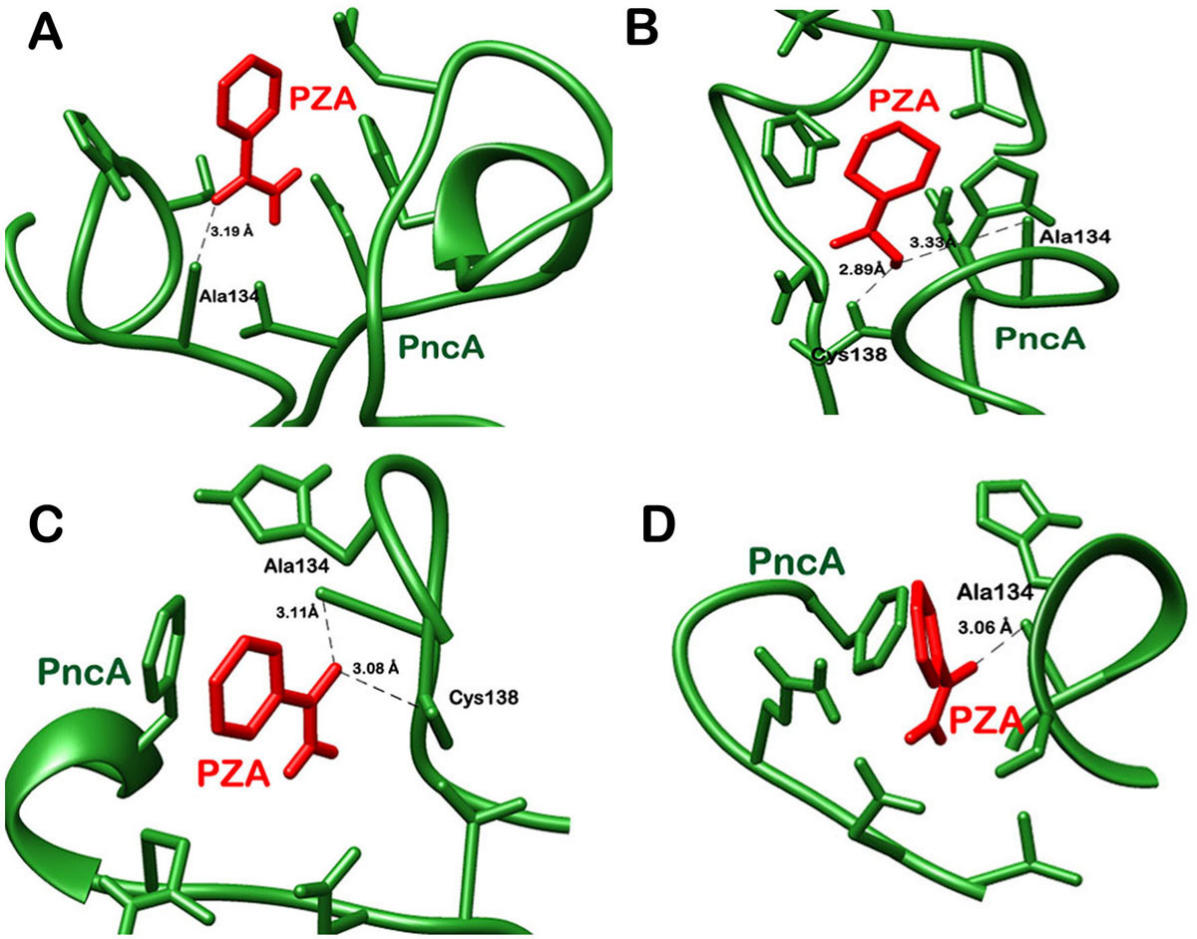
**Depiction of hydrogen bond interactions in A) native protein bound to PZA after docking, B) native protein bound with PZA after MD simulations, C) docked mutant PncA-PZA complex, D) mutant protein bound to PZA post MD simulations**.

**Figure 3:**
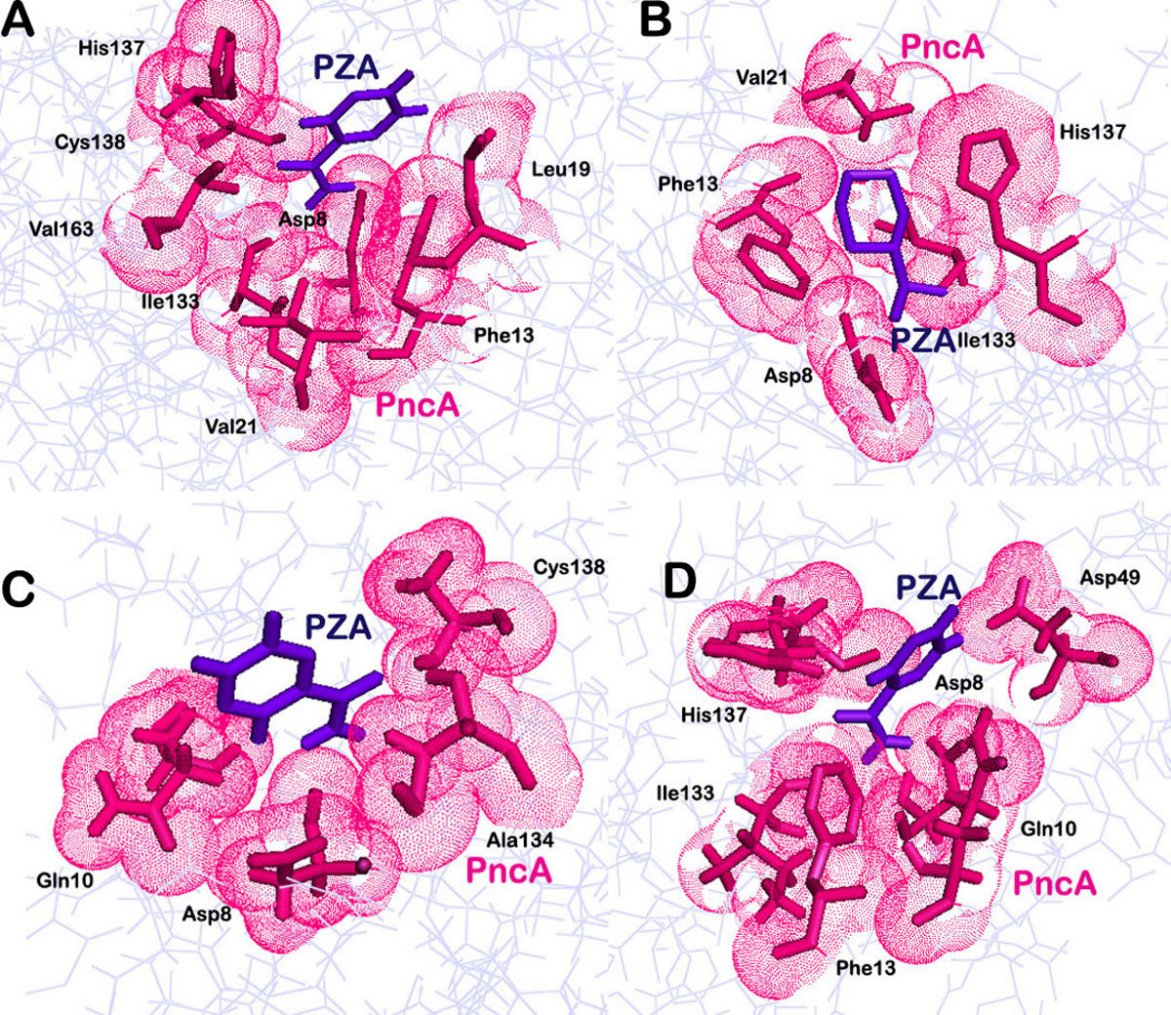
**Hydrophobic interaction pattern in A) native protein bound to PZA after docking, B) native protein bound with PZA after MD simulations, C) docked mutant PncA-PZA complex, D) mutant protein bound to PZA post MD simulations**.

In native complex, an oxygen atom of PZA was found making one hydrogen bond with the nitrogen of Ala 134 of PncA and the distance was 3.19Å (Figure 2A). The ligand also made a significant number of hydrophobic bonds with Asp 8, Phe 13, leu1 9, Val 21, Ile 133, His 137, Cys 138 and Val 163 of PncA (Figure 3A). On the other hand the mutant protein was found to be making two hydrogen bonds. An oxygen atom of the ligand was making hydrogen bonds with nitrogen of Ala 134 and Cys 138 of mutated protein and the bond lengths were 3.11 Å and 3.08 Å respectively (Figure 2C). However, the number of residues involved in making hydrophobic contacts was less in the case of mutated protein-PZA complex. Amino acids of mutated PncA participating in hydrophobic interactions included Asp 8, Gln 10, Ile 133 and His 137 (Figure 3C). We observed a difference in the interaction pattern of the two proteins with PZA. Cys 138, one of the key active residues was involved in hydrophobic interaction in native protein complex while in mutant complex it was forming a hydrogen bond with the ligand. Over all, PZA had a lower binding affinity towards the mutated form of PncA as indicated by the docking score of -3.77 in comparison to the wild type protein, with which it had a glide docking score of -4.025. To further validate the binding affinity two online servers, PatchDock and SwissDock were also used. The results obtained indicated a lower affinity of PZA for the mutated protein in comparison to the wild type. It was in coherence with the results obtained using Glide.

All the three factors, namely, volume of binding pocket, docking score and interaction energy, typically used here to explain the reason for resistance to drug supported the idea of weak binding of the ligand with the protein in mutated form, which was hampering its activation. However, increased number of hydrogen bonds in mutant complex was suspicious. Therefore, in order to study the conformational changes occurring in the complexes in dynamic environment similar to *in vivo *conditions, we performed molecular dynamics simulation studies. Calculation and analysis of parameters like RMSD, radius of gyration and RMSF was done to understand the behavior of binding of the ligand with the enzyme.

### Investigation of flexibility of native and mutant PncA enzyme

**Figure 4:**
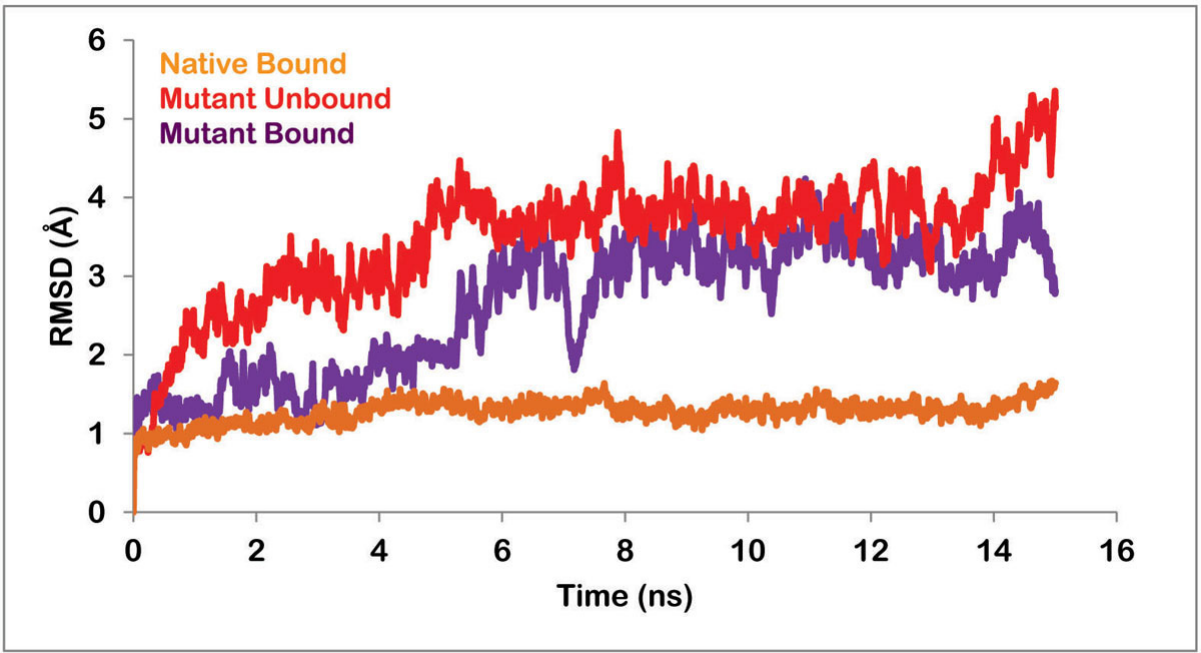
**Graph showing the RMSD of backbone of native unbound protein, native bound to PZA, mutant protein in unbound form and mutant protein in complex with PZA**.

PZA bound native and mutant proteins were simulated in a SPC water box for around 15 ns. RMSD trajectory was analyzed for both the complexed systems. As shown in Figure 4, the native unbound and native protein complex, both were quite stable. The native unbound display low fluctuations and remain stable throughout the simulations. The native bound also did not deviate much from its initial docked conformation. However, in the mutant structure, the protein complex showed deviation up to 3Å in the first half phase of the simulation run and then gave a stable RMSD trajectory (Figure 4).

Some changes were observed in the interaction pattern of the native and mutant complexes post molecular dynamics studies. In native complex, the number of hydrogen bonds increased to two. The oxygen atom of PZA was making two hydrogen bonds with nitrogen of Ala 134 and Cys 138 with bond lengths of 3.33Å and 2.89Å respectively (Figure 2B). The hydrophobic interactions however reduced. PZA was now making hydrophobic contacts with Asp 8, Phe 13, Val 21, Ile 133 and His 137 of native PncA (Figure 3B). On the other hand, mutant complex was now making only one hydrogen bond with Ala 134 (Figure 2D). The bond length for the corresponding hydrogen bond was observed to be 3.06Å. Seven amino acid residues of mutant proteins, namely Asp 8, Gln 10, Phe 13, Asp 49, Ile 133, His 137 and Cys 138 were now interacting hydrophobically with PZA (Figure 3D).

**Figure 5:**
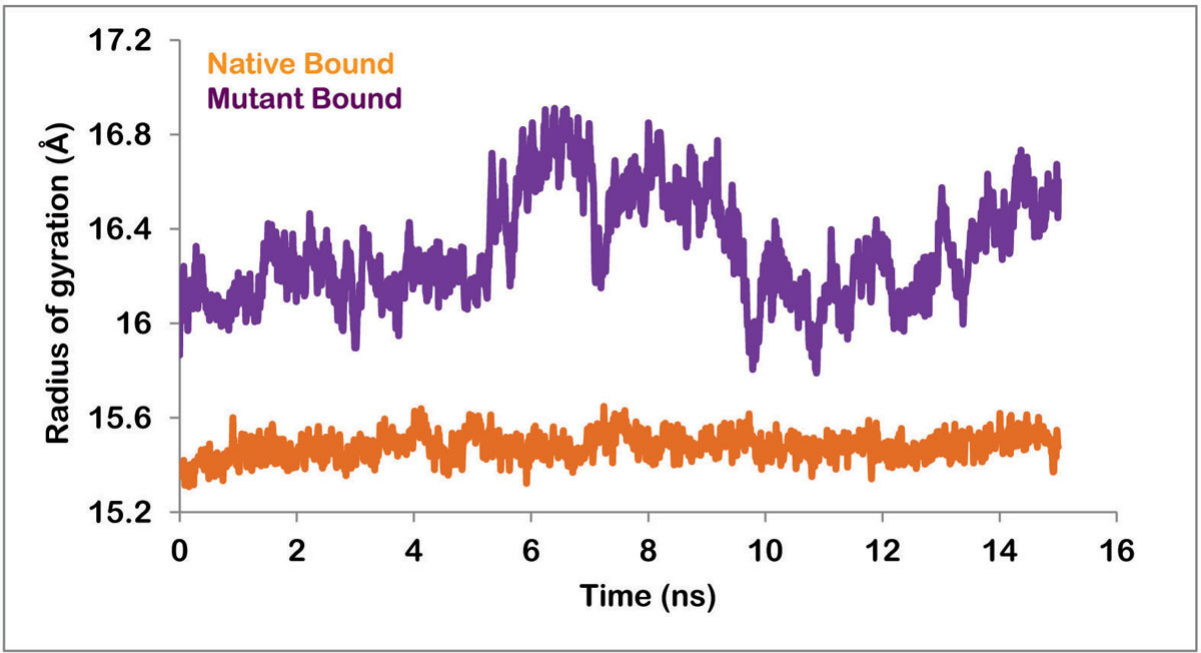
**Radius of gyration of native and mutant protein in complex with PZA**.

The radius of gyration is defined as the mass-weighted root mean-square distance of a cluster of atoms from their common center of mass. In other words, the radius of gyration of a protein is a measure of its compactness. A protein will maintain a steady value of *R_g _*if it is stably folded, and the value will fluctuate if the protein unfolds. The radius of gyration of all the frames during the simulation run was plotted against time and the data was analyzed. The curve for the native complex was stable throughout the simulation period and fluctuations were negligible. This steady curve can be attributed to high stability and compactness of the protein. The plot for the mutant protein complex was however more fluctuating and the value for R_g _reached as high as 17 Å (Figure 5). This fluctuation was prevalent during the entire simulation period. Hence it can be inferred that the protein was losing its compactness due to the change in its conformation.

**Figure 6:**
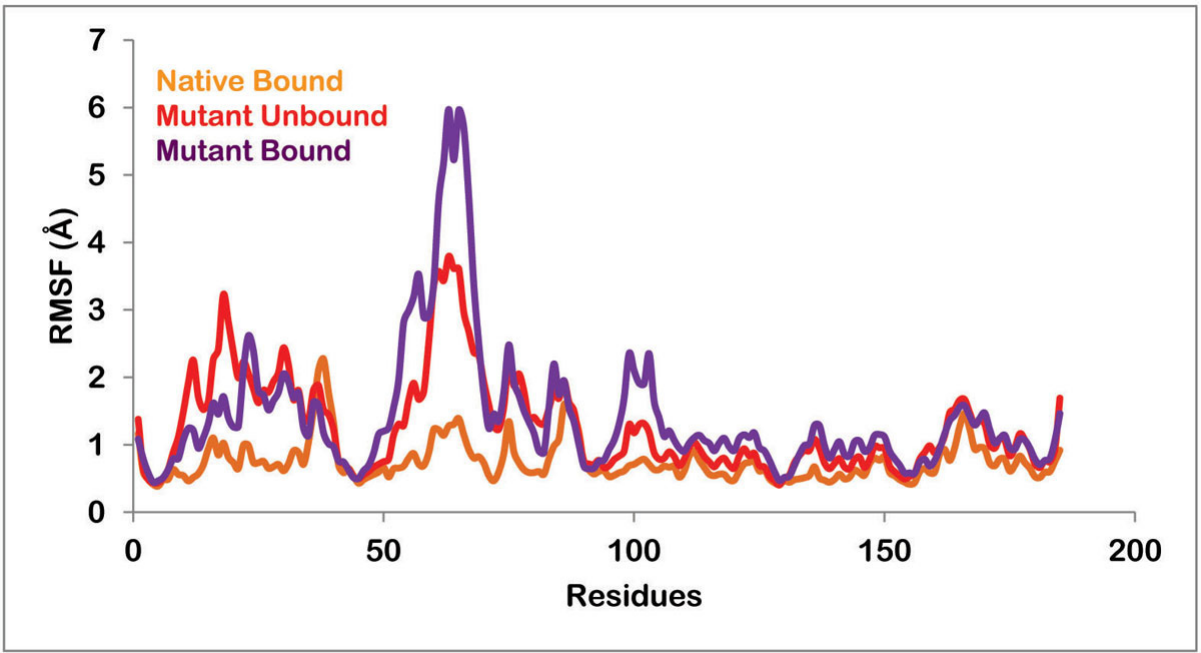
**Root mean square fluctuation of all the residues in native protein bound to PZA, mutant protein in unbound form and mutant protein in complex with PZA**.

Root mean square fluctuation (RMSF) with respect to each residue in both native and mutant complex was then calculated to explain the loosening of the mutant protein structure. The fluctuations were widespread in the mutant protein, while in native sequence small fluctuations were observed in certain regions. The fluctuations in certain residues of native protein were conferring flexibility to the protein and keeping it compact at the same time. While in case of mutant protein, high level of fluctuations depicting high mobility of the residues was resulting in a loosely packed protein. As illustrated in Figure 6, fluctuations were highest in the loop region i.e. from 51 to 71 amino acid residues. This result was in accordance with information obtained from superposition shown in Figure 1. The loop region of the mutated protein moved away from the active cleft, which was also the reason behind increase in the volume of catalytic cavity. According to a study, residues 51 to 71 compose an important loop region or a flap which is involved in holding the ligand in position after binding. Thus this loosening of the loop region was affecting the binding of PZA with the active residues.

The RMSF of catalytically important binding residues, Asp 8, Phe13, IIe133, Ala134 and Cys138 of native and mutant structures were also analyzed. Table 2 lists the RMSF values of all these residues in unbound mutant, mutant and native protein bound with PZA. The fluctuation in binding residues was very small in case of native protein while these residues were highly flexible in case of mutant protein in both bound and unbound form.

**Table 2:** RMSF values of binding cavity residues

Protein	Asp 8	Phe 13	Ile 133	Ala 134	Cys 138
Native (Bound)	0.635117	0.572594	0.496256	0.5135	0.476051
Mutant (Unbound)	0.890527	1.71058	0.84011	0.91584	0.731542
Mutant (Bound)	0.795579	0.948946	0.858351	0.99094	0.988013

All these analyses brought the discussion to the point that in mutated form the flap region of the protein becomes more flexible and moves away from the active site. This results in a much bigger active cavity. Once the ligand, PZA comes and binds to the active residues, flap is not able to hold it in bound state thereby preventing the activation of the prodrug.

Previously studies have been performed on PncA mutations namely, D8G, S104R and C138Y. The analysis showed that the binding cavity volume decreased and the cavity became rigid. This did not permit proper alignment of PZA inside the cavity and hampered hydrogen bond formation. Our study included a novel mutation. The mutation being present in the flap region, affected the volume of the cavity in contrast to the previous mutations. The mutation led to a substantial increase in the binding cavity. This prohibited the enzyme from holding the drug properly and therefore PZA could not take its active form [24].

## Conclusion

This study is intended to present a comprehensive analysis of the drug resistance mechanism caused by K96R mutation in PncA enzyme of *M. tuberculosis*. Here we have performed binding pocket analysis, molecular docking and molecular dynamics simulation studies. The binding cavity analysis showed a significant increase in the volume of the active cavity from 551.9 to 1314.2 Å^3 ^upon mutation. This fact was further supported by RMSF, radius of gyration and simulations studies. We showed that the flap region becomes highly flexible in mutated protein and moves away from the active cavity and is no longer able to hold the bound drug in its place. This prevents the activation of the prodrug to this functional form. And hence the patient with this K96R mutation develops resistance to PZA drug. This study gives insight into the mechanism by which drug resistance is acquired and therefore can be helpful while designing new drugs for tuberculosis treatment. Using the analysis, new classes of drugs can be produced which have a better conformation such that the binding to important residues is not affected by the changing volumes. Ligands should possess functional groups which permit the establishment of strongly interacting hydrogen bonds with the binding residues.

## Competing interests

The authors declare that they have no competing interests.

## Authors' contributions

CV, NB and AG designed the methods and experimental setup. CV, JKD, SG and AnG carried out the implementation of the various methods. CV, JKD, SG and AG wrote the manuscript. All authors have read and approved the final manuscript.

## Declaration

AG would like to acknowledge financial support from Jawaharlal Nehru University towards publication of this article.

This article has been published as part of *BMC Genomics *Volume 16 Supplement 2, 2015: Selected articles from the Thirteenth Asia Pacific Bioinformatics Conference (APBC 2015): Genomics. The full contents of the supplement are available online at http://www.biomedcentral.com/bmcgenomics/supplements/16/S2
